# Investigating social inequalities in children’s independent mobility, active transportation and outdoor free play in two Canadian cities

**DOI:** 10.1016/j.pmedr.2024.102642

**Published:** 2024-02-05

**Authors:** Zeinab Aliyas, Patricia A. Collins, Marie-Pierre Sylvestre, Katherine L. Frohlich

**Affiliations:** a Centre de recherche en santé publique (CReSP), Université de Montréal, Montréal, Québec, Canada; b Department of Geography and Planning, Queen’s University, Kingston, Ontario, Canada; c Département de médecine sociale et préventive, École de santé publique de l’Université de Montréal, Montréal, Québec, Canada; d Centre de recherche du centre hospitalier de l’Université de Montréal (CRCHUM), Montreal, QC, Canada

**Keywords:** Free play, Active transportation, Independent mobility, Socioeconomic status, Children’s physical activity, Cities

## Abstract

**Background:**

Active transportation (AT) and free play (FP) are the primary ways in which children engage in unstructured physical activity in cities, with independent mobility (IM) gaining increased attention as a potential precursor of AT and FP. However, current trends show that children are engaging in less FP and AT, and have less IM, than previous generations and it is not well understood how these practices, and their interrelatedness, differ by neighbourhood-level socio-economic stats (SES) and municipal contexts

**Objectives:**

This study aims to address the gaps in knowledge by quantifying, comparing, and correlating IM, AT, and FP practices in high and low-SES neighbourhoods within and across the cities of Montreal and Kingston, Canada

**Methods:**

584 questionnaires were distributed among children in grades 1 to 5, living in low- and high-SES neighbourhoods of these two cities

Results

Engagement in the three practices was low in every study neighbourhood, though all three practices were higher in high-SES compared to low-SES neighbourhoods in both cities. Levels of FP were higher in Kingston compared to Montreal, while AT was higher in Montreal than in Kingston

**Conclusion:**

This study revealed social inequalities in all three of these practices based on socioeconomic status and city. Since IM is likely a precursor to both independent FP and AT, more research is warranted into how our cities can become more conducive to IM in children, particularly in low SES neighbourhoods where children have less freedom of movement independently and otherwise.

## Highlights

• Engagement in children’s Free Play, Independent Mobility, and Active Travel was generally low.
• Children residing in higher SES neighborhoods exhibited higher levels of these activities.
• Children in Montreal showed a higher rate of Active Travel compared to Kingston.
• Children living in Kingston demonstrated a higher level of Free Play compared to Montreal.

## 1 Introduction

Automobile-centric urban planning has profoundly limited children's active and independent mobility in city environments over the last century (Carroll et al., 2015). This transformation has been fueled by safety-focused intensive parenting practices, an overdependence on automobiles, and urban designs that prioritize motorist convenience (Tremblay et al., 2015). This pattern contributes to reducing the presence of children on streets and heightening risks for pedestrians and cyclists across all age groups (Collins and Kearns, 2005, Tranter and Pawson, 2001). As a result, children's unstructured physical activities have relocated to areas perceived to be safe, like backyards and parks, altering the social and physical landscape of their play (Lee et al., 2016, Moran et al., 2017, Veitch et al., 2006). This shift away from public spaces reshapes not only where children play but also the social dynamics influencing their active lifestyles (Faulkner et al., 2015, Janssen, 2015). Such conditions significantly curtail children’s engagement in unstructured physical activity, notably seen in the declining trend of children's independent mobility (IM) – their ability to walk around their neighborhoods independently or with peers within a reasonable distance from home (Glanz et al., 2008).

Like IM, free play (FP) is an essential part of childhood development (Loebach et al., 2021). Free play can occur in a private space, such as a yard, or a public space like a street, park, or school yard, either in the company of other children or alone. However, the scarcity of secure and conveniently accessible outdoor areas has contributed to the decline of FP among children (Barnes and Tremblay, 2016). A study by Moran et al. (2017) found that only 17 % of children reported playing in the streets, and Lee et al. (2016) found that over 60 % of children play outdoors for fewer than 2 h in a typical week.

Meanwhile, the prioritization of automobile-oriented mobility has been blamed for the widespread decline in children's use of active transportation (AT) (Hillman, 1990, Larouche et al., 2020, van der Ploeg et al., 2008). This, in turn, has hindered children from actively traveling (by walking or cycling) to various destinations such as schools and parks. AT, especially to school, can provide opportunities for children to engage in regular physical activity and reduce their reliance on automobiles (Toner et al., 2021). However, the lack of safe infrastructure for AT has posed barriers for children to walk or cycle to school or other destinations within their communities, further contributing to a reduction in their physical activity levels (Carver et al., 2008a).

Neighbourhoods can provide valuable resources and/or present harmful exposures that profoundly impact child development and well-being, and many of these vary by socio-economic status (SES) (Aarts et al., 2012, Davison and Lawson, 2006, Galobardes, 2006). As such, it is critical to explore the link between neighbourhood-level SES and children’s unstructured physical activity levels. While previous research has demonstrated social inequalities in children’s IM and engagement in AT and FP (Bianchi et al., 2008, Mutz and Albrecht, 2017), studies exploring the association between the socioeconomic level of neighbourhoods and children's engagement in these practices are limited, with inconsistent results (Carver et al., 2014, Delisle Nyström et al., 2019, Larsen et al., 2009, McDonald, 2008, Veitch et al., 2007, Ziviani et al., 2008). Some studies have shown that children living in lower SES neighbourhoods face greater barriers to FP and AT (Larsen et al., 2015, Loebach et al., 2021, Veitch et al., 2017). These children may have restricted access to safe and accessible play spaces, which can limit their opportunities for unstructured play and exploration (Larsen et al., 2015). Additionally, the lack of reliable and affordable transportation options can further hinder their ability to engage in activities outside their immediate neighbourhood (Veitch et al., 2017). These findings suggest that children from lower SES neighbourhoods may face challenges in accessing the same level of freedom of movement and play opportunities as their peers from higher SES backgrounds.

On the other hand, it is important to consider that SES is complex and multidimensional and its effects can vary across and between cities. In some instances, lower SES children may exhibit a greater degree of freedom and access to transportation (Stone et al., 2014). For example, in certain low-income neighbourhoods, children may have more unstructured time, which they fill with outdoor play time (Kimbro et al., 2011). Furthermore, these children might rely more on AT, such as walking or cycling, due to limited access to private vehicles or public transportation (Fyhri et al., 2011).

The decline of children's IM, AT, and FP internationally over the last 20–30 years has been influenced by multiple factors (Glanz et al., 2008, Lambert et al., 2019). Although previous studies have suggested that IM can provide opportunities for children to engage in more FP and AT (Frohlich and Collins, 2024, Moran et al., 2017, Schoeppe et al., 2013, Stone et al., 2014, Veitch et al., 2007), it is not well known how these practices vary by neighbourhood-level socio-economic context and city context. And, while researchers have drawn links between IM and FP (Veitch et al., 2007), and IM and AT (Delisle Nyström et al., 2019), rarely are these practices studied together, with the links between them poorly understood. As such, this study aims to address these shortcomings in the literature by: 1) quantifying the levels of IM, AT, and FP among children residing in high- and low-SES neighbourhoods in Montreal, Quebec, and Kingston, Ontario; 2) investigating how IM, AT, and FP differ across neighbourhood-level SES within each city and within neighbourhood-level SES between cities; and 3) measuring the correlation between IM, AT, and FP by neighbourhood-level SES within each city. A more nuanced understanding of how children’s engagement in IM, AT, and FP varies by neighbourhood and city context is needed for practitioners and policymakers seeking to employ equity-informed measures to improve children's health, well-being, and independence.

## 2 Methods

### 2.1 Study design

This paper reports on baseline data collected from a population health intervention study entitled *Levelling the Playing Fields*. The purpose of this comparative case study was to conduct a realist evaluation of the implementation and outcomes associated with two street closure interventions – Play Streets and School Streets – that were being pilot tested in high and low-SES neighbourhoods in the two Canadian cities of Montreal, Quebec, and Kingston, Ontario. Baseline data were collected from participating and control neighbourhoods prior to the launch of the street closure interventions (summer/autumn 2021 and summer/autumn 2022). Ethics approval was granted by the research ethics boards of the respective universities at each study site. All research participants, including the children and their parents/guardians, provided informed consent and assent. The study met each institution's guidelines for protection of human subjects concerning safety and privacy.

### 2.2 Municipality profiles

Participants for this study were drawn from socio-economically contrasting neighbourhoods of Montreal and Kingston. These cities were chosen to maximize learning for the pilot study, as they provided multiple sources of differentiation (i.e., city size, socio-demographics, provincial jurisdiction, and culture) that could influence the implementation and outcomes associated with the school closure interventions being tested. According to the latest Census (Statistics Canada, 2022), the city of Montreal is roughly 10 times larger than the city of Kingston in terms of population and population density, and Montreal has more than double the proportion of first-generation immigrants and visible minorities when compared to Kingston. Meanwhile, the city of Kingston has notably higher median household income and rates of high school completion compared to Montreal (Table 1). The selection of Montreal and Kingston was also supported by strong partnerships with community partners in each city that were committed to the implementation of the street closure interventions for the duration of the research study.

**Table 1:** Socio-demographic portrait of the cities of Montreal and Kingston, based on 2021 Census Subdivision Areas (CSD).

	**Montreal**	**Kingston**
Population, 2021	1,762,949	132,485
Population density per square kilometre	4834	293
Median after-tax income of household in 2020 ($)	56,000	70,500
% Immigrants	33	14
% Recent immigrants*	6	2
% Visible minority population	39	13
% No high school diploma or equivalency**	12	7

Source: 2021 Canadian Census, Statistics Canada.

*Recent immigrant refers to a person who obtained a landed immigrant or permanent resident status up to five years prior to a given Census year.** For the population aged 25 to 64 years in private households.

### 2.3 Neighbourhood selection and participant recruitment

Sampling and recruitment of study participants began at the neighbourhood level. Intervention and control neighbourhoods were selected based on two criteria. First, we selected neighbourhoods with high proportions of children under 14 years of age relative to the city average, since this was the target population for the street closure interventions. Second, we selected neighbourhoods of high and low socioeconomic status to facilitate comparisons of intervention implementation and outcomes. In each city, statistics for child population and socioeconomic status drew from the 2016 Canadian Census. For Kingston, the socioeconomic indicator used to select neighbourhoods was the 2015 median after-tax household income at the Census Tract level, as large disparities were observable across neighbourhoods in Kingston based on household income alone. As there is a greater degree of income mix at the neighbourhood level in Montreal, the Montreal team relied on the Material Deprivation Index – a composite of income, education and employment variables from the Census - to select neighbourhoods that fell into the highest and lowest quintiles of deprivation (Pampalon and Guy, 2000). In Montreal, a total of seven neighbourhoods were selected, comprising three neighbourhoods of lower SES and four neighbourhoods of higher SES. In Kingston, six neighborhoods were chosen, consisting of three high-SES and three low-SES neighbourhoods.

### 2.4 Questionnaire administration and design

Data were collected through parent–child paired questionnaires with children from grades 1 to 5. Our objective was to encompass a minimum of 60 children within each neighborhood, ensuring a balanced representation of girls and boys across diverse grade levels. Originally, the plan was to collect all data online in both cities. However, challenges faced in certain low-SES neighborhoods in Montreal prompted the team to explore alternative data collection methods to ensure the required participant numbers were reached. To achieve this, we employed various approaches for survey recruitment and administration in both school and residential settings to accommodate different preferences and levels of accessibility across communities.

In Kingston, recruitment of survey participants was two-pronged: hard copy invitations were delivered to all residents’ mailboxes within the selected neighbourhoods; and study notices were shared through geographically targeted advertising on Facebook. Parents and children who were interested in participating were instructed to email PC to enrol in the study. All questionnaires from Kingston were self-administered online.

In Montreal, recruitment of survey participants included delivery of hard-copy invitations to residents’ mailboxes, as well as study notices sent directly from schools, within selected neighborhoods. Survey administration involved a hybrid approach of self-administered online surveys and hard-copy questionnaires, with the latter administered by members of the research team.

Child questionnaires were completed by children, with the assistance of their parent/guardian when required. The child questionnaire was organized into three sections capturing children’s IM, FP, and AT. Children’s age and gender were documented in the accompanying parent questionnaire. The data collection tools were pilot tested to confirm their face validity.

#### 2.4.1 Independent mobility

We utilized a previously validated mobility licenses’ scale to gather information from children (Hillman, 1990). These licenses include six items: permission for children to carry out activities on their own such as crossing and cycling on main roads, taking local buses, going out after dark, traveling home from school, and visiting other places within walking distance. The licenses were categorized as either yes (1) or no (0) and the values were summed to obtain an IM index ranging from 0 to 6. Higher scores on the IM index indicate greater independent mobility. The scale has been found to have acceptable test–retest reliability in a pilot study (Larouche et al., 2017), with intraclass correlation coefficients (ICC) of 0.76 and 0.77 based on answers provided by both the child and their parent.

#### 2.4.2 Outdoor free play

A scale validated by Veitch et al. (Veitch et al., 2009), and adapted by Janssen (Janssen, 2015), was used to ask children about the frequency of their outdoor play in various locations during a typical week in the past month. The scale consisted of seven items corresponding to the following locations where children play: the yard at their home, the yard at someone else's home (friend, neighbour or relative), the street or cul-de-sac their home is on, other streets or cul-de-sacs, parks and playgrounds outside of school hours, school grounds outside of school hours, and other places where they can be active (e.g., field, parking lot, forested area). There are six response options for each of these items with scores ranging from 0 to 7: never/rarely (0), less than once a week (0.5), 1–2 times per week (1.5), 3–4 times per week (3.5), 5–6 times per week (5.5), and daily (7). The scores from the seven items are added to create an outdoor play frequency score with a potential range from 0 to 49. The test–retest reliability of these items has been assessed using ICC, which ranged from 0.58 to 0.82 (Veitch et al., 2009).

#### 2.4.3 Active transportation

An active transportation scale developed by Carver et al. (Carver et al., 2008b) and Timperio et al. (Timperio, 2004) and adapted by Janssen (Janssen, 2015) asked children about the frequency with which they walked or bicycled to various locations during a typical week over the past month. Eight locations were included, consisting of: school; home; the homes of friends, neighbours, or relatives; sports venues; parks and playgrounds; convenience/variety stores; fast food restaurants or coffee shops; and other shops and destinations (e.g., malls or community centers). There are six response options for each of the items, with scores ranging from 0 to 7: never/rarely (0), less than once a week (0.5), 1–2 times per week (1.5), 3–4 times per week (3.5), 5–6 times per week (5.5), and daily (7). A potential score range of 0 to 56 for the frequency of active transportation was created based on the scores from the eight items. The test–retest reliability of these items was determined to have an ICC of approximately 0.5 (Carver et al., 2008b).

### 2.5 Statistical analyses

The data was analyzed using SPSS version 25. The first step in the analysis was to examine the demographic information of the children, including their gender and age. Descriptive statistics were used to analyze these data, and the results were reported based on the frequency expressed as a percentage. This analysis provided an overview of the sample and was used to identify trends that might exist among participants in different study neighbourhoods. Next, the mean, median, and standard deviation (SD) for FP, AT, and IM were calculated and presented by low- and high-SES neighbourhoods in each city. Multiple linear regression analysis was employed to assess the potential associations of each outdoor activity with neighborhood-level socioeconomic status and cities. In each model, gender and age were included as control variables. Residual analysis was conducted to evaluate the fit of the models. Finally, Spearman correlations were used to explore the relationship among the three activities (OFP, AT, and IM) in high and low SES neighbourhoods across the two cities.

## 3 Results

The total sample size was 537 (381 participants from Montreal and 156 from Kingston). Incomplete questionnaires were excluded (n = 47; 37 from Montreal and 10 from Kingston) if the child responded to fewer than 30 % of the questions. Table 2 summarizes the gender and age profile of participating children based on neighbourhood-level SES in Montreal and Kingston. Despite some minor variations, participants were roughly evenly split by gender and age grouping.

**Table 2:** Descriptive statistics of children's gender and age across montreal and kingston neighbourhoods (2021–2022).

	**Montreal**		**Kingston**	
	**Low-SES neighbourhoods (n = 160)**	**High-SES neighbourhoods (n = 221)**	**Low-SES neighbourhoods (n = 68)**	**High-SES neighbourhoods (n = 88)**
	**Number (%)**			
**Gender**				
**Girls**	87 (56.9)	104 (49.1)	29 (42.6)	46 (52.9)
**Boys**	66 (43.1)	108 (48.9)	38 (55.9)	41 (47.1)
**Missing data**	7 (4.4)	9 (4.1)	1 (1.5)	1 (1.1)
**Age**				
**6**–**7**	55 (34.6)	52 (24.5)	16 (25.8)	32 (36.4)
**8**–**9**	58 (36.5)	82 (38.7)	20 (32.3)	31 (35.2)
**10**–**12**	46 (28.9)	78 (36.8)	26 (41.9)	25 (28.4)
**Missing data**	1 (0.6)	9 (4.1)	6 (8.8)	0

Children’s engagement in FP, AT, and IM are presented in Table 3. The scores were extremely low for all three practices in every neighbourhood in both cities. Comparisons within cities revealed that children’s engagement in all three practices (FP, AT, IM) was higher in high-SES neighbourhoods compared to low-SES neighbourhoods. Between cities, FP was highest in high-SES neighbourhoods in Kingston, followed by low-SES neighbourhoods in Kingston; AT was highest in high-SES neighbourhoods in Montreal, followed by low-SES neighbourhoods in Montreal; and IM was highest in the high-SES neighbourhoods of both Montreal and Kingston.

**Table 3:** Descriptive statistics for FP, AT and IM based on neighbourhood-level SES in Montreal and Kingston (2021–2022).

	**Free Play***Values range 0 to 49*	**Active Transportation***Values range 0 to 56*	**Independent Mobility***Values range 0 to 6*
	**Low-SES neighbourhoods in Montreal (n = 160)**		
Mean (SD)	9.20 (6.41)	13.83 (10.03)	0.85 (1.40)
Median	7.5	14	0
Minimum	0	0	0
Maximum	35	49	6
	**High-SES neighbourhoods in Montreal (n = 221)**		
Mean (SD)	12.59 (6.86)	17.18 (11.09)	1.30 (1.52)
Median	12	16	1
Minimum	1	0	0
Maximum	33	49	6
	**Low-SES neighbourhoods in Kingston (n = 68)**		
Mean (SD)	14.27 (7.89)	10.12 (8.32)	0.97 (1.33)
Median	13.75	7.5	0
Minimum	2.5	0	0
Maximum	38	31	5
	**High-SES neighbourhoods in Kingston (n = 88)**		
Mean (SD)	21.42 (9.47)	14.33 (9.46)	1.29 (1.48)
Median	20.50	13.25	1
Minimum	3.5	0	0
Maximum	47.5	38.5	5

Multiple linear regression models were conducted to explore the relationship between neighborhood-level SES and children's outdoor activity while controlling for age and gender. Prior to this analysis, the residuals did not reveal any issues with the model fit. The examination of neighborhood-level SES in Montreal indicated a positive association of high neighborhood-level SES with both FP and AT. In Kingston, a significant positive association between high neighborhood-level SES and all three practices was observed. When comparing these activities across the two cities, significantly higher levels of FP were found in high-SES neighbourhoods in Kingston compared to high-SES neighbourhoods in Montreal. The same pattern was observed in low-SES neighbourhoods, with higher levels of FP reported in Kingston compared to Montreal. In contrast, AT was significantly higher in both high-SES and low-SES neighbourhoods of Montreal compared to Kingston. Notably, there were no significant relationships with IM across the two cities (see Table 4).

**Table 4:** The relationship of FP, AT and IM with SES in two cities; Montreal and Kingston (2021–2022).

**Montreal (High SES vs Low SES)**			
Unstandardized coefficient (95 % CI)			
	**Free Play**	**Active Transport**	**Independent Mobility**
**SES**	3.21 (1.81, 4.62)**	3.02 (0.77, 5.27)**	0.22 (−0.40, 4.81)
**Gender**	1.56 (0.18, 2.94)*	1.70 (−0.50, 3.91)	0.09 (−0.16, 0.34)
**Age**	0.47 (0.42, 0.90)*	0.80 (0.12, 1.48)*	0.52 (0.44, 0.60)**
**Kingston (High SES vs Low SES)**			
Unstandardized coefficient (95 % CI)			
	**Free Play**	**Active Transport**	**Independent Mobility**
**SES**	8.08 (5.20, 10.96)**	4.5 (1.57, 7.44)*	0.41 (0.003, 0.83)*
**Gender**	0.12 (−2.66, −2.91)	−0.71 (−3.55, −2.13)	0.01 (−0.38, 0.41)
**Age**	0.36 (−0.41, 1.13)	0.66 (−0.12, 1.44)	0.38 (0.27, 0.49)**
**Montreal vs Kingston (Low SES)**			
Unstandardized coefficient (95 % CI)			
	**Free Play**	**Active Transport**	**Independent Mobility**
**City**	4.09 (20.09, 6.10)**	−4.34 (−7.12, − 1.55)**	0.06 (−0.30, 0.43)
**Gender**	−0.004 (−1.80, − 1.80)	1.88 (−0.61, 4.39)	0.02 (−0.31, 0.35)
**Age**	0.42 (−0.07, 0.93)	1.13 (0.43, 1.84)**	0.37 (0.28, 0.46)**
**Montreal vs Kingston (High SES)**			
Unstandardized coefficient **(95 % CI)**			
	**Free Play**	**Active Transport**	**Independent Mobility**
**City**	8.92 (6.96, 10.87)**	−2.75 (−5.47, −0.41)*	0.29 (−0.01, 0.60)
**Gender**	1.95 (0.18, 3.72)*	0.04 (−2.40, 2.50)	0.13 (−0.15, 0.41)
**Age**	0.46 (−0.96, 1.02)	0.30 (−0.46, 1.07)	0.60 (0.47, 0.65)**

^**^P <.01., * P <.05.

Table 5 summarizes the Spearman correlation coefficients for the three variables (AT, FP, and IM) among children residing in high and low-SES neighbourhoods of Montreal and Kingston. Positive correlations between FP and AT, FP and IM, and AT and IM were found in all neighbourhoods. We found moderate correlations (>0.300) between AT and IM in low-SES neighbourhoods in Kingston, low correlations in Montreal, and moderate association between AT and FP in high-SES neighbourhoods in both cities.

**Table 5:** Correlation between AT, FP, and IM based on neighbourhood-level SES in Montreal and Kingston.

**Low-SES neighbourhoods in Montreal**		
	**Active Transport**	**Independent Mobility**
**Free Play**	0.202	0.156
**Active Transport**		0.291
**High-SES neighbourhoods in Montreal**		
	**Active Transport**	**Independent Mobility**
**Free Play**	0.411	0.275
**Active Transport**		0.284
**Low-SES neighbourhoods in Kingston**		
	**Active Transport**	**Independent Mobility**
**Free Play**	0.308	0.171
**Active Transport**		0.426
**High-SES neighbourhoods in Kingston**		
	**Active Transport**	**Independent Mobility**
** Free Play**	0.419	0.126
**Active Transport**		0.121

Spearman correlation (2-tailed) was performed to assess the correlation between variables.

## 4 Discussion

Children’s engagement in free play, active transportation, and independent mobility profoundly contribute to their overall physical activity levels (Pabayo et al., 2012, Razak et al., 2018, Schoeppe et al., 2014). And yet, little is known about how these practices, and their interrelatedness, vary across contexts. Thus, the objective of this research was to analyze these three practices among children in low- and high-SES neighbourhoods in two Canadian cities.

First and foremost, this study found that children’s engagement in all three practices was low, regardless of neighbourhood-level SES or city of residence. This finding aligns with previous studies highlighting limitations in children's engagement in unstructured physical activity and active mobility (Glanz et al., 2008; Riazi et al., 2021).

Secondly, our study found consistently lower levels of engagement in all three practices among children residing in low-SES neighbourhoods, aligning with previous studies in this field (Larsen et al., 2015, Loebach et al., 2021, Veitch et al., 2017). These findings emphasize the importance of focusing on interventions and policies specifically tailored to low-SES neighbourhoods to directly address the barriers faced by these children (Lahelma, 2006). Previous research has shown that children and their parents in low-SES neighbourhoods experience multiple barriers to enabling children’s engagement in unstructured physical activities, including scheduling demands, financial barriers, family obligations, and environmental barriers (e.g. lack of sports facilities and playgrounds, safety issues) (Brockman et al., 2009, Holt et al., 2011, Humbert et al., 2006, Veitch et al., 2013). It is therefore crucial to implement changes and strategies based on the specific needs and characteristics of each community to promote children’s independence and activity more effectively.

The third substantial finding from this study was the higher levels of FP in Kingston and AT in Montreal. These differences can likely be attributed to distinct urban design features, as well as other social and physical characteristics, that differentiate the two cities. Kingston's lower population and dwelling density and the prevalence of backyards likely contribute to the higher engagement in FP observed among children, as previous research has indicated that private yards are the most common place for children to engage in play (Veitch et al., 2010). Meanwhile, the higher population density and sophistication of AT-supportive infrastructure in Montreal likely explain the higher levels of AT, given the importance of these factors for AT (Kontou et al., 2020).

Finally, the correlations observed between FP, AT, and IM suggest that promoting one activity can have positive effects on the others. Policies and practices should focus on creating supportive environments that encourage children's IM, AT, and FP in unison. For example, promoting IM can lead to increased opportunities for FP and AT. Similarly, creating safe and accessible AT options can enhance children's IM and FP experiences. Considering the interconnections between these activities, theoretical frameworks can guide the development of comprehensive strategies addressing multiple aspects of children's physical activity and overall well-being.

Factors such as urban design, density, public policy, and cultural attitudes shape the opportunities and constraints for children's engagement in IM, AT, and FP (Carver et al., 2008a, Ding et al., 2011, Schoeppe et al., 2014, Shaw et al., 2013). Exploring the reasons behind these differences can provide insights for designing effective interventions at the municipal level, tailored to the specific needs and characteristics of each city. Cities with extensive pedestrian infrastructure, low traffic volumes, and supportive policies can facilitate greater engagement in these activities among children (Krishnamurthy, 2019, Mitra et al., 2021). Additionally, variations in neighbourhood safety and community support for outdoor play have important roles in shaping children's unstructured physical activity-related behaviours (Heath and Bilderback, 2019, Kyttä et al., 2018).

To address the issue of low levels of these practices among children, several recommendations can be made. First, creating safe and accessible street spaces will be crucial to stimulating outdoor free play and independent mobility. This can involve implementing traffic calming measures, creating pedestrian-friendly infrastructure, and designating play streets and school streets where children can engage in more IM and activities of all forms (Carver et al., 2008a, Hawley et al., 2018, Mitra and Abbasi, 2020). Second, promoting active transportation options, such as walking or cycling to school, can increase children's physical activity levels (Denstel et al., 2015). This can be achieved through the development of safe walking and cycling routes and the implementation of initiatives like school streets, walking school buses, or bike trains (Buliung et al., 2011, Scharoun Benson et al., 2020, Wilson et al., 2019).

This study has several limitations. Firstly, acknowledging self-selection bias is vital, where parents more interested in the subject may have skewed study participation, potentially affecting the results. Secondly, variations in data collection methods across cities could contribute to disparities in our results. Different techniques might have influenced responses, introducing inconsistencies in comparisons between cities. Thirdly, reliance on recall measures pose risks of social desirability and self-reporting biases, impacting data accuracy. In addition to these considerations, it's crucial to examine household-level factors, especially how household income impacts children's outdoor activities. This approach would contribute to a more comprehensive understanding of the influence of socioeconomic factors at different scales (neighbourhood and household levels). To address these limitations, future research should employ random sampling, standardize data collection methods, and integrate objective measures alongside self-reports to ensure comprehensive analysis. Future studies should also explore various area-level factors—physical and social—linked to neighborhoods when assessing children's outdoor activities and their potential influence on differences in various socio-economic neighborhoods.

This study contributes to our understanding of the relationship between children's engagement in IM, AT, and FP, as well as how these practices can vary by neighbourhoods and city contexts. By emphasizing the widespread limitation of children's mobility and activity, regardless of neighborhood context, the study highlights the urgent need for comprehensive interventions targeting these essential activities. Moreover, it sheds light on the differences in engagement levels between different socioeconomic neighborhoods, calling for tailored strategies to overcome barriers prevalent in low-SES areas.

## 5 Conclusion

Findings from this study reveal concerningly low levels of IM, AT, and FP among children across all studied neighbourhoods. These practices play a crucial role in shaping children's physical activity, social development, and overall health and well-being. It is crucial for researchers and practitioners to prioritize promoting IM, AT, and FP, particularly in low-SES neighbourhoods. By creating safe environments and providing opportunities for FP, AT, and IM, we can support children in leading healthier and more active lives.

## 6 Institutional review board statement

The study was conducted in accordance with the Declaration of Helsinki, and approved by the General Research Ethics Board of Queen’s University (protocol 6029274, approved August 19, 2020) and by the Comité d’éthique de la recherche en sciences et en santé (CERSES) (Projet 2020-908, approved June 30, 2021).

## 7 Informed consent statement

Informed consent was obtained from all subjects involved in the study.

## 8 Disclosure statement

This work was supported by funding from the Canadian Institutes of Health Research (Grant Number 175153, awarded on March 25, 2021, to KLF and PC). One of the authors (PC) resides in one of the high SES Kingston neighbourhoods that was selected for this study and knows some of the survey respondents from this neighbourhood personally.

## Funding

This research was funded by the Canadian Institutes of Health Research (grant number 175153 (2021-03-25) to KLF and PC). MPS is also a recipient of the Junior 2 Salary Award from the Fonds de recherche du Québec - Santé (FRQS).

## CRediT authorship contribution statement

**Zeinab Aliyas:** Writing – review & editing, Writing – original draft, Resources, Methodology, Formal analysis, Conceptualization. **Patricia A. Collins:** Conceptualization, Funding acquisition Data curation, Writing – original draft, Writing – review & editing, Validation, Supervision, Formal analysis. **Marie-Pierre Sylvestre:** Conceptualization, Data curation, Writing – original draft, Writing – review & editing, Validation, Formal analysis. **Katherine L. Frohlich:** Conceptualization, Funding acquisition Data curation, Writing – original draft, Writing – review & editing, Validation, Supervision, Formal analysis.

## Declaration of competing interest

The authors declare that they have no known competing financial interests, and three of the four authors have no personal relationships, that could have appeared to influence the work reported in this paper. One of the authors (PC) resides in one of the high SES Kingston neighbourhoods that was selected for this study, and knows some of the survey respondents from this neighbourhood personally.

## Data Availability

Data will be made available on request.
